# Computational Analysis and Predictive Cheminformatics Modeling of Small Molecule Inhibitors of Epigenetic Modifiers

**DOI:** 10.1371/journal.pone.0083032

**Published:** 2016-09-13

**Authors:** Salma Jamal, Sonam Arora, Vinod Scaria

**Affiliations:** 1 CSIR Open Source Drug Discovery Unit (CSIR-OSDD), Anusandhan Bhawan, Delhi, India; 2 Delhi Technological University, Delhi, India; 3 GN Ramachandran Knowledge Center for Genome Informatics, CSIR Institute of Genomics and Integrative Biology (CSIR-IGIB), Delhi, India; University of Nevada School of Medicine, UNITED STATES

## Abstract

**Background:**

The dynamic and differential regulation and expression of genes is majorly governed by the complex interactions of a subset of biomolecules in the cell operating at multiple levels starting from genome organisation to protein post-translational regulation. The regulatory layer contributed by the epigenetic layer has been one of the favourite areas of interest recently. This layer of regulation as we know today largely comprises of DNA modifications, histone modifications and noncoding RNA regulation and the interplay between each of these major components. Epigenetic regulation has been recently shown to be central to development of a number of disease processes. The availability of datasets of high-throughput screens for molecules for biological properties offer a new opportunity to develop computational methodologies which would enable in-silico screening of large molecular libraries.

**Methods:**

In the present study, we have used data from high throughput screens for the inhibitors of epigenetic modifiers. Computational predictive models were constructed based on the molecular descriptors. Machine learning algorithms for supervised training, Naive Bayes and Random Forest, were used to generate predictive models for the small molecule inhibitors of histone methyl-transferases and demethylases. Random forest, with the accuracy of 80%, was identified as the most accurate classifier. Further we complemented the study with substructure search approach filtering out the probable pharmacophores from the active molecules leading to drug molecules.

**Results:**

We show that effective use of appropriate computational algorithms could be used to learn molecular and structural correlates of biological activities of small molecules. The computational models developed could be potentially used to screen and identify potential new biological activities of molecules from large molecular libraries and prioritise them for in-depth biological assays. To the best of our knowledge, this is the first and most comprehensive computational analysis towards understanding activities of small molecules inhibitors of epigenetic modifiers.

## Introduction

Though all cells in an organism inherit the same genomic template, the dynamic expression of the genome provides for the cell-type and tissue specific organisation and functional organisation of multi-cellular organisms [1]. This dynamic regulation is largely dependent on the regulatory layer of interactions between multiple biomolecules, operating at the chromatin organisation, transcriptional and post-transcriptional levels [2]. The regulatory layer contributed by the Epigenetic layer has been one of the favourite areas of interest recently [3]. The epigenetic layer of regulation comprises largely of DNA modifications, histone modifications and noncoding RNA regulation and the interplay between each of these major components. The understanding of this epigenetic layer of gene regulation has largely been fuelled by large-scale genome-wide maps of both DNA modifications and histone modifications [4], thanks to the availability of high-throughput sequencing based assays to qualify epigenetic marks across the genome. Epigenetic modifications and their dysregulation has been implicated in the pathophysiology of a wide spectrum of diseases [3].Though present understanding of the role of epigenetic dysregulation contributing to the pathophysiology of diseases is rudimentary, a number of diseases including cancers [5] neuropsychiatric disorders [6], metabolic disorders [7] have been shown to have a strong association with epigenetic dysregulation.

Histone organization and post-transcriptional modification of histones contribute a major and well studied class of epigenetic marks. Histone proteins are integral components of the nucleosome and post-transcriptional modification of histones and their interplay with DNA base modifications largely regulate the transcription of genes. These post-transcriptional modifications of histones are modulated by proteins popularly known as histone modifiers, which dynamically regulate the pattern of modifications across the genome through a concerted, but poorly understood mechanism. Ample evidence in the recent years have shown that DNA methylation and histone modifications could modulate gene expression [8], mark gene boundaries [9] and potentially differentiate between protein-coding and noncoding gene promoters in the genome [10,11]. Histone modifiers or Epigenetic modifiers are largely categorised into three groups [12]. The first group of proteins largely post-translationally ‘write’ marks on the histone tail. Well studied examples of such proteins include histone Methyltransferases or acetyltransferases. The second group of proteins largely ‘erase’ existing marks on the histone, and include well characterized proteins like demethylases and deacetylases. The third and potentially poorly understood class of proteins recognise specific epigenetic marks and bind to the histone complex modulating their regulatory effect.

Epigenetic modifiers have been recently studied in detail as they could be attractive drug targets for diseases where epigenetic dysregulation play an important role, as in the case of some cancers [13]. This has been complemented by the availability of high throughput screening methodologies and assays for many of these proteins [14, 15]. The availability of these large-scale screening datasets in public domain also provide an immense opportunity to model the activities based on physicochemical and/or structural properties of molecules. These models would be immensely useful in drug discovery applications to significantly reduce the time and effort by prioritising molecules with desirable activities for in-depth screening and biological validation. Such approaches have been extensively used by our group previously towards modelling activities [16–19].

In the present study, we employed four datasets of high-throughput screens for inhibitors of epigenetic modifiers. We used machine learning approaches using chemical descriptors to create accurate predictive models for the activities. We also use an independent substructure based approach to identify commonly enriched substructures associated with the activities. To the best of our knowledge, this is the first report of computational modelling of biological activities of small molecule inhibitors of epigenetic modifiers.

## Methodology

### Dataset source

The data for the potential inhibitors of Histone methyltransferases and demethylases was downloaded from PubChem repository of chemical compounds. The datasets were downloaded corresponding to AID 504332, AID 504339, AID 2147 and AID 540317.

#### Bioassay AID 504332

The qHTS was based on an assay developed for the inhibitors of G9a (Histone Lysine Methyltransferase) and included 30,875 active and 2, 67,000 inactive compounds. G9a is a histone methyltransferase which belongs to SET-domain containing family and specifically catalyzes methylation of Lys9 of histone H3 (H3K9) in mammalian euchromatic regions repressing the transcription [20, 21]. The knockdown of G9a results in transcriptional activation and inhibits cancer cells growth [22].

#### Bioassay AID 504339

The dataset contains inhibitors of JMJD2A-Tudor Domain JMJD2A, which is a jumonji-domain-containing histone demethylase (Lysine-specific demethylase 4A). JMJD2A binds to trimethylated H3K4 and H4K20 via the tudor domains and causes demethylation which may result in both, transcriptional repression and activation [23, 24]. Binding of JmjD2A to histone results in positioning of the enzymes for methylating adjacent regions causing rapid methylation over large area of chromatin [25, 26]. Targeting of the JMJD2A-tudor domain interaction with the methylation marks on lysine residues of histone, H3 and H4, tails may lead to selective demethylation of a given methyllysine locus based on the methylation state of adjacent histone marks. The demethylase activity follows radical attack mechanism using Fe (II) and α-ketoglutarate as co-factors. The substrate (mono, di or tri-methylated lysine) to be demethylated is determined by the association of enzymes with cofactors. The data contain 16,919 active compounds and 3, 38,945 inactive compounds.

#### Bioassay AID 2147

The dataset contains small molecule inhibitors of Human Jumonji Domain Containing 2E (JMJD2E). JMJD2E is a histone modifier enzyme and functions as a histone demethylase. Histone lysine demethylases catalyze the demethylation of methylated lysine side-chains on histones H3 and H4, thus acts to reverse the methylation reactions catalyzed by histone lysine methyltransferases. The high throughput data contained a total of 3,523 active and 1, 88,950 inactive compounds.

#### Bioassay AID 540317

The assay was developed to identify the first inhibitors of protein methyltransferases. The dataset contained 2,142 active and 3, 67,962 inactive compounds screened for potential inhibitors of HP1-beta chromodomain interactions with Methylated Histone Tails HP1 (Heterochromatin protein). The N- terminal chromodomain containing HP1 proteins bind to the methylated histones and further results in gene repression and heterochromatin formation. The interaction harbors an N- terminal chromodomain that binds to the tri-methylated lysine 9 of histone H3, H3K9me3, and a C-terminal chromoshadow domain. Compounds in PubChem are characterized based on Activity Score calculated using AC50. AC50 is the concentration at which 50% of the activity is observed. Compounds having AC50 values less than or equal to 20 micromolar with corresponding activity score between 40–100 were considered as active compounds. Compounds having AC50 value greater than the highest concentration tested (for example 20 micromolar) and activity score 0 were considered as inactive compounds. The rest compounds with activity score between 1–39 were considered as inconclusive compounds.

All the datasets were obtained through the confirmatory bioassay screens conducted by NCGC, NIH Molecular Libraries Probe Production Network. The Amplified Luminescent Proximity Homogeneous Assay (AlphaScreen) from PerkinElmer was used for identification of these inhibitors. It is a homogeneous assay technology used for screening of different classes of targets and analytes. Donor and acceptor beads coated with a layer of hydrogel are utilized. The beads are conjugated with biological molecules. With excitation, ambient oxygen is converted to reactive singlet oxygen in the donor bead. The singlet oxygen species reacts with thioxene compounds in the acceptor bead to generate a chemiluminescent signal that emits at 370 nm. Streptavidin-coated and anti-IgG antibody-coated beads were employed for detecting the methylation state of biotinylated-histone peptide.

### Pre-processing of data

The files obtained from PubChem in the Structure Data Format (SDF) were used to generate 2D molecular descriptors using PowerMV [27], a popular toolkit for computing chemical descriptors. PowerMV generated a total of 179 molecular descriptors describing the physicochemical properties of the molecule (like hydrogen bond donors, acceptors, number of rotatable bonds, charge, polarizability, aromaticity etc.). The descriptor file generated was saved in comma separated (CSV) format. The attributes having same bit string descriptor values throughout the dataset (all 0’s or all 1’s) were filtered out. The list of the descriptors generated and used is available as S1 Table.

### Processing data and model building

The files saved in CSV format were then converted to ARFF (Attribute Relation File Format) compatible with Weka. The models were built using different classifications viz. Naive Bayes and Random Forest as described previously [16, 17, 18, 19]. For each base model we have used cost sensitive classifier in order to reduce the false negative rate. This was imperative as the datasets were highly imbalanced with a large inactive set compared to the active set. The misclassification cost is incremented on false negatives until the false positive rate reduces to 20%. Assignment of the cost is random irrespective of the base classifier used. Cost sensitive classification is based on cost-proportionate weighting of the training examples, which can be realized either by applying weights to the classification algorithm, or by resampling of instances according to costs. We used Weka 3.6 (Waikato Environment for Knowledge Analysis) [28] which is a toolbox for data mining and machine learning algorithms. Weka provides a simple GUI supporting data from various sources and in different file formats. It has multiple algorithms (including that of regression, association rule mining, clustering, classification etc.) and pre-processing tools that allow comparison of different methods. The workbench is used for both supervised as well as unsupervised algorithms. The data visualization facilities help in easy access and analysis of results. The dataset was then further randomly split into training and test sets. The training data comprising maximum part of complete data (80%) was used to train the model using different algorithms and the test set (20%) was to evaluate each of the models. All computation was performed on CDAC-Garuda supercomputing facility using the OSDD-Garuda web interface.

### Cross validation

K-fold cross validation is one of the most popularly sued methods of cross-validation of the accuracy of a model. In k-fold cross validation, the entire data is divided into k subsets (folds) of equal sizes and training is done for (k-1) sets and testing is done on one set. The process is repeated k number of times so that each set is tested at least once. The average error rate is computed for all tests. We have used (k = 5) or a 5-fold cross validation here since the dataset was large.

### Model performance evaluation

The 2X2 confusion matrix used by Weka contains the following values:

- True positives (TP): class members classified as class members.
- True negatives (TN): class non-members classified as non-members.
- False positives (FP): class non-members classified as class members.
- False negatives (FN): class members classified as class non-members.

We additionally used the following measures for the statistical evaluation of the models:

- **Sensitivity** is the proportion of actual positives which are predicted positive, i.e.TP / (TP + FN).
- **Specificity** is the proportion of actual negatives which are predicted negatives, i.e. TN / (TN + FP).
- **ROC** is receiver operating characteristic curve which is a 2D curve parameterized by one parameter of the classification algorithm, e.g. some threshold in the true positive rate /false positive rate.
- The **Matthews correlation coefficient** is a measure of correlation between the observed and predicted binary classifications and quality of the classifier. The values range between −1 and +1.
- **Balanced Classification Rate (BCR)** introduces a balance in the classification calculated as 1⁄2. (Sensitivity + Specificity).
- **Accuracy** is the efficiency of the classifier to predict true values, i.e. TP+TN) / (TP+TN+FP+FN) * 100).

### Substructure search

Aligning structures on a key structural framework help chemists to interpret the influence of substituents on the template as initial point towards understanding structure-activity information. With the aim of finding molecules which have similar properties to act as a drug, similarity search of compounds was done. Library MCS, a tool from ChemAxon [29], based on hierarchical clustering algorithm was used to cluster the molecules and find the potential bioactive substructures. Hierarchical clustering only requires a measure of similarity between groups of data points. Maximal Common Substructure Search (MCS) is the process of finding the largest structure that is a substructure or part of all the molecules in a given set. Initial structures are found at the bottom of the hierarchy. The next level contains the maximum common structures of clusters of initial molecules; subsequent levels provide larger clusters of smaller common substructures.

Minimal MCS size refers to the smallest size of the maximum common substructure searched for by the algorithm. For different datasets different values were considered owing to the number of top level clusters found and the level count. For AID 504332 and 540317, minimal MCS size was taken 9 and for AID 504339 and 2147 it was 10 and 11 respectively. After the clusters were formed using LibMCS, we got the molecular scaffolds in the form of sdf and SMILES file. The active and inactive 3D structure files were then used to search the similar substructures with the smiles generated. This was done using the jcsearch algorithm of ChemAxon [30]. The similarity is calculated on the basis of the molecular descriptor or fingerprint of the chemical structures to compare. The Substructures were evaluated for enrichment using chi-square test. The p-values were computed to evaluate the significance of enrichment. The substructures which had at least 1% matches among the active dataset entries, p-value less than 0.01 and enrichment factor more than 5 were considered significant.

## Results

The datasets obtained from PubChem were processed to generate 2D molecular descriptors using PowerMV. The descriptors were finally culled to 155 from 179 descriptors after removing values which were either null or the same for the entire dataset and could not contribute to the classification (S1 Table). The complete data after splitting into train and test sets was loaded in Weka-3.6 to build different classifier models for the evaluation of compounds. Initially standard classification of the data was performed. However, since the datasets were skewed, cost sensitive classification was introduced. The misclassification cost was applied on false negatives and incremented until the rate of false positives reached 20%. The costs applied in different datasets for different models are shown in Table 1. Naive bayes used minimum cost for the classification of the objects.

**Table 1:** Misclassification costs used for False Negatives (FN).

*PubChem Assay ID*	*Naive Bayes*	*Random Forest*
**AID 504332**	2	50
**AID 504339**	10	1000
**AID 2147**	45	3000
**AID 540317**	30	25000

**Fig 1:**
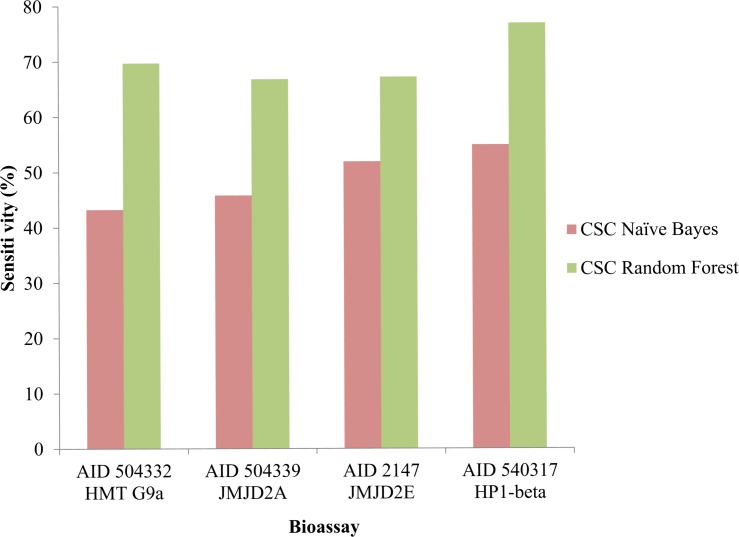
Graph between sensitivity of Naive Bayes and Random Forest amongst AID 504332, 504339, 2147 and 540317.

**Fig 2:**
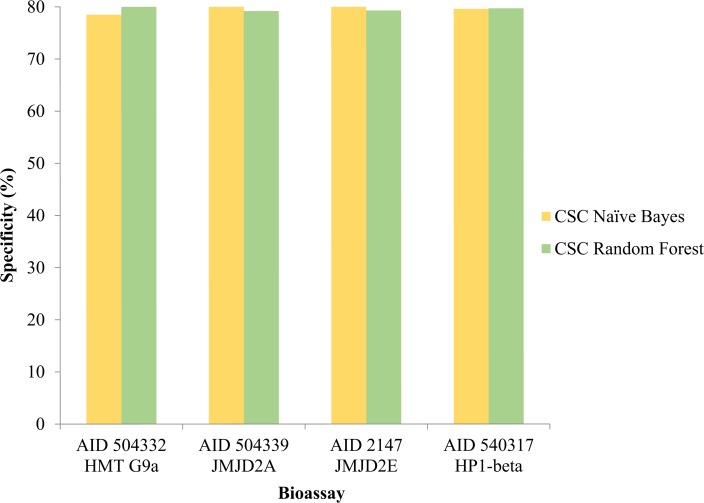
Comparative graph between the specificity of both classification models amongst all four datasets.

Evaluation of models included various statistical parameters. The accuracy of Random forest was predicted to be the highest for all the datasets. A comparison between the sensitivity of both the classification models amongst different datasets was made depicting the sensitivity of Random forest more than the Naive Bayes in all cases. Recent studies on comparison of classifiers performance have reported that Random forest outperforms Naïve bayes in terms of accuracy of prediction [31]. Random forest uses multiple decision trees to place an instance into a particular class and the output is the average of the individual trees output whereas Naïve bayes is a probabilistic approach which calculates the probability of a compound being in a particular class on the basis of presence of certain independent features [32]. It has also been shown that the assembly of trees gives much accurate prediction than single one [33]. Our group has also previously shown that the Random forest has outperformed Naïve bayes in similar cheminformatics analyses [16–19] on bioassay datasets. Fig 1 shows the plot between sensitivity of Naive Bayes and Random Forest amongst AID 504332, 504339, 2147 and 540317. Similarly the specificity was compared where Naive Bayes outperforms in AID 504339 and 2147. In case of AID 540317 specificity of both was comparable and Random Forest showed higher specificity in AID 504332. Fig 2 is the comparative graph between the specificity of both classification models amongst all four datasets.

The sensitivity and specificity were used to calculate the balanced classification rate for each model. Random forest showed the most balanced classification out of both. As a measure of quality, Matthews’s correlation coefficient (MCC) was calculated. The Matthews correlation coefficient (MCC) also known as the phi coefficient is a measure of correlation between the actual and predicted classifications. Table 2 shows the classification results of all the datasets along with the statistical evaluation. The summary of the parameters are summarised in Table 3.

**Table 2:** Overall summary of the performance of the models.

*PubChem Assay ID*	*Classifier*	*TP Rate*	*FP Rate*	*ROC*	*Accuracy*	*MCC*	*BCR*
***AID 504332***	CSCNB	43.2	21.5	66.5	74.84	0.15	60.89
	CSCRF	69.7	19.5	82.1	79.39	0.35	75.11
***AID 504339***	CSCNB	45.8	19.6	68.5	79.43	0.10	63.08
	CSCRF	66.8	20.8	79.4	78.88	0.17	72.99
***AID 2147***	CSCNB	51.9	20.0	72.4	79.58	0.09	65.97
	CSCRF	67.2	20.7	80.1	79.12	0.14	73.24
***AID 540317***	CSCNB	54.9	20.4	74.2	79.45	0.06	67.25
	CSCRF	76.9	20.3	85.8	79.67	0.10	78.28

**Table 3:** Shows the details of the LibMCS cluster report for different data sets studied.

LibMCS parameters	AID 504332	AID 504339	AID 2147	AID 540317
MCS size	9	10	11	9
Level Count	6	6	5	6
Top level cluster count	726	1026	702	216
Total Cluster Count	5150	7113	3791	1437
Singletons	258	416	365	93
Significant substructures	19	9	9	8

**Fig 3:**
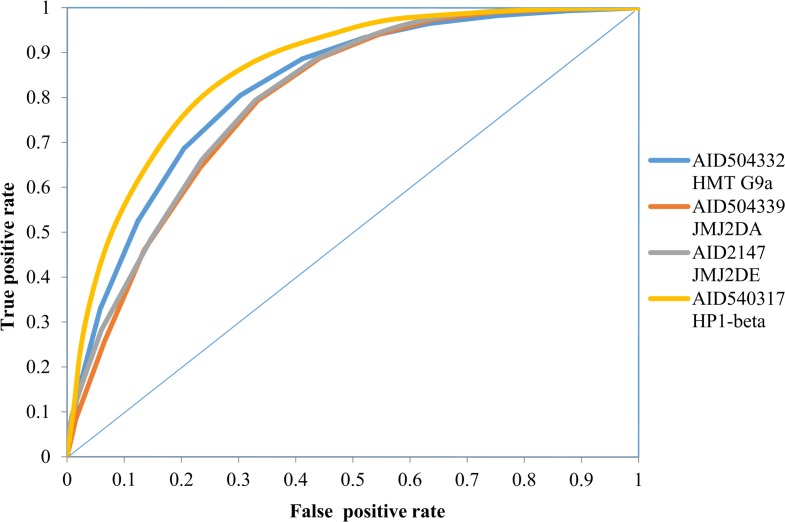
ROC plot for Random Forest classification model for the four datasets.

A perfect test would have 100% sensitivity and specificity, but in realistic scenarios, however this is seldom achieved and a balance between sensitivity and specificity is desirable. For that, a relation of sensitivity and specificity on a graph, called a "Receiver-Operator-Characteristic (ROC) curve" was plotted. Fig 3 summarises the ROC plot for Random Forest classification model for the four datasets. The Area under the curve for the ROC-plots was 0.82, 0.68, 0.80 and 0.67 for the AID 504332, AID 504339, AID 2147 and AID 540317 respectively.

### Evaluation of significantly enriched scaffolds

In the process of drug discovery the local similarity between the structures proved to be useful in designing of new chemical compounds as potential drugs. We used JChem module, LibMCS and clustered the active compounds of all the datasets. In the present study, we have also evaluated enriched substructures, rather than substructures which are exclusive for the actives. Conceptually this would mean that the feature/substructure in consideration alone would not have necessary predictive ability to define activity, but rather has a larger probability to be found among the actives. It is believed that the compounds having similar structures often have similar properties and biological activities [34].Our analysis shows a subset of the substructures are significantly enriched in the actives compared to inactives by a few folds. The presence of the feature/substructure alone would not have necessary predictive ability to define activity, but rather has a larger probability to be found among the actives.

### Clustering analysis of AID 504332

The 30875 active compounds clustered into a total of 5,150 clusters of which the 726 top level cluster compounds were considered. The compounds were clustered upto level 6 out of which 258 singletons were removed. The enrichment and its significance, was analyzed by chi-square test. Analysis revealed 19 significantly enriched scaffolds which had p-value less than 0.01 and an enrichment factor > 5.

### Clustering analysis of AID 504339

A total of 16919 active compounds were clustered upto 6 levels at MCS size 10. 416 singletons were removed and 1026 compounds obtained at top level were taken for further analysis. We obtained 9 substructures prioritized by p-value (less than 0.01) and enrichment factor > 5.

### Clustering analysis of AID 2147

The 3523 compounds were clustered keeping MCS size as 11, we obtained 3791 total clusters. A total of 702 compounds were obtained at level 5 out of which 365 singletons were removed. The final prioritization was done keeping p value less than 0.01 and enrichment factor > 5, the analysis resulted in 9 substructures.

### Clustering analysis of AID 540317

The 2142 active compounds were clustered upto 6 levels keeping MCS size as 9. We obtained 216 compounds at top level after removing 93 singletons. Analysis revealed 8 significantly enriched scaffolds which had p-value less than 0.01 and an enrichment factor > 5.

The results for the clustering approach used and the enriched scaffold for each of the datasets considered have been summarised in tables (S2–S5 Tables).

## Discussion

Understanding the function and regulation of epigenetic modifier proteins have been recently an actively pursued area of research [35]. This has been more so, with the increasingly understood mechanisms of epigenetic regulation in the pathophysiology of a number of diseases. The role of epigenetic modifiers has been extensively studied in a variety of neoplasms [36, 37, 38, 39, 40]. It has also been discussed that molecules that could target epigenetic modifiers could be a potential new avenue for drug development [41]. In fact, targeting epigenetic modifiers as potential drug targets have been extensively discussed and pursued [42, 43]. The cornerstone of any rational drug discovery process starts from systematic screening of molecular libraries against target proteins, and assaying them for their biological outputs or phenotypes. Testing large libraries of molecules for specific biological activities are usually time consuming and extremely costly. Computational methods for pre-selecting molecules from large libraries would offer a plausible time and cost-effective alternative [41]. It has been suggested that accurate methodologies to pre-select molecules for in-depth biological assays would accelerate the process of drug discovery. A number of methodologies including molecular docking [44, 45] and other cheminformatics methods [46, 47, 48] have been extensively used to prioritise molecules in drug discovery process. Machine learning approaches have been used extensively now for building predictive models for pre-selecting molecules form large molecular databases [16–19]. The availability of datasets of high-throughput screens on large molecular libraries of small molecules which are quite diverse offers an enormous opportunity to learn molecular and structural properties of molecules and their association or correlation with phenotypic or biological outcomes.

The present manuscript discusses how computational methods could be used to mine potential actives based on chemical descriptors. We propose a computational pre-screening would improve the chance of finding an active from a given dataset, which could potentially imply savings in cost and resources. In real-life situations, chemists synthesize molecules based on scaffolds which are largely associated with activity. We have additionally used a substructure enrichment analysis to understand substructures which are largely associated with activity, nevertheless does not imply activity. This would allow chemists to suitably modify molecules or be able to synthesize molecules of a particular variety or based on a particular scaffold, so as to potentially improve the hit rate. We do find a significant large number, albeit a significant small number by proportion due to the very fact that there are a far higher number of molecules in the inactive set compared to that of actives.

We show that machine learning based approaches can provide computational models which are highly accurate which could be potentially used to screen large molecular libraries. The study is not without caveats, the first being the paucity of data sets in public domain encompassing inhibitors for a large number of epigenetic modifiers precludes us from creating a comprehensive suite of predictive models, which could be eventually possible with more data sets being available in public domain. In the future, many such computational models could be integrated to provide for desirable set of properties or biological activities and has the potential to be integrated into drug discovery pipelines, with significant gains in the cost and timespan associated with a conventional drug discovery process [49]. The present study also provides the first comprehensive overview and cheminformatics analysis of small molecule modulators of epigenetic modifiers.

## Supporting Information

S1 Table List of the molecular descriptors used from PowerMV. (DOCX)

S2 Table Depicts significantly enriched scaffolds found in AID 504332. (DOCX)

S3 Table Shows the significant substructures found in AID 504339 along with their p-value and chi-square statistics. (DOCX)

S4 Table Shows the enriched substructures in AID 2147 with a threshold of 5 for enrichment factor and p-value less than 0.01. (DOCX)

S5 Table Shows significantly enriched substructures in AID 540317. (DOCX)
