# Supplementary material for: Computational Analysis and Predictive Cheminformatics Modeling of Small Molecule Inhibitors of Epigenetic Modifiers
Source: PLoS One. 2016 Sep 13;11(9):e0083032. doi: 10.1371/journal.pone.0083032 (PMC5021286; doi:10.1371/journal.pone.0083032)
Supplement: S1 Table — (DOCX) [file pone.0083032.s001.docx]

**Supplementary Table 1.** List of the molecular descriptors used from PowerMV.

| Pharmacophore  Fingerprint | Weighted Burden Number | Property |
| --- | --- | --- |
| NEG_01_NEG- NEG_07_NEG  NEG_03_POS-NEG_07_POS  NEG_01_HBD-NEG_07_HBD  NEG_03_HBA-NEG_07_HBA  NEG_02_ARC-NEG_07_ARC  NEG_02_HYP-NEG_07_HYP  POS_03_POS-POS_07_POS  POS_02_HBD-POS_07_HBD  POS_03_HBA-POS_07_HBA  POS_02_ARC-POS_07_ARC  POS_02_HYP-POS_07_HYP  HBD_03_HBD-HBD_07_HBD  HBD_03_HBA-HBD_07_HBA  HBD_02_ARC-HBD_07_ARC  HBD_02_HYP-HBD_07_HYP  HBA_03_HBA-HBA_07_HBA  HBA_03_ARC-HBA_07_ARC  HBA_02_HYP-HBA_07_HYP  ARC_01_ARC-ARC_07_ARC  ARC_02_HYP-ARC_07_HYP  HYP_01_HYP-HYP_07_HYP | WBN_GC_L_0.25, WBN_GC_H_0.25,  WBN_GC_L_0.50, WBN_GC_H_0.50,  WBN_GC_L_0.75, WBN_GC_H_0.75,  WBN_GC_L_1.00, WBN_GC_H_1.00,  WBN_EN_L_0.25, WBN_EN_H_0.25,  WBN_EN_L_0. 50, WBN_EN_H_0. 50,  WBN_EN_L_0.75, WBN_EN_H_0.75,  WBN_EN_L_1.00, WBN_EN_H_1.00,  WBN_LP_L_0.25, WBN_ LP _H_0.25,  WBN_ LP _L_0. 50, WBN_ LP _H_0. 50,  WBN_ LP _L_0.75, WBN_ LP _H_0.75,  WBN_ LP _L_1.00, WBN_ LP _H_1.00 | XLogP, PSA, NumRot,  NumHBA, NumHBD, MW,  BBB, BadGroup |
