# Supplementary material for: Computational Analysis and Predictive Cheminformatics Modeling of Small Molecule Inhibitors of Epigenetic Modifiers
Source: PLoS One. 2016 Sep 13;11(9):e0083032. doi: 10.1371/journal.pone.0083032 (PMC5021286; doi:10.1371/journal.pone.0083032)
Supplement: S2 Table — (DOCX) [file pone.0083032.s002.docx]

**S2 Table:** Depicts significantly enriched scaffolds found in AID 504332.

| **Scaffold No.** | **Scaffold Structure** | **Matches in Actives** | **Matches in Inactives** | **Chi- square** | **p-value** | **Enrichment**  **Factor** |
| --- | --- | --- | --- | --- | --- | --- |
| 1 | 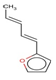 | 54 | 8 | 392.99 | 1.85E-87 | 58.372 |
| 2 | 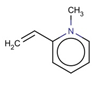 | 45 | 9 | 309.521 | 2.78E-69 | 43.239 |
| 3 | 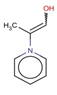 | 50 | 15 | 309.998 | 2.19E-69 | 28.826 |
| 4 | 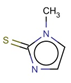 | 39 | 14 | 228.039 | 1.60E-51 | 24.090 |
| 5 | 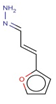 | 31 | 14 | 165.918 | 5.77E-38 | 19.149 |
| 6 | 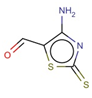 | 44 | 29 | 195.765 | 1.75E-44 | 13.121 |
| 7 | 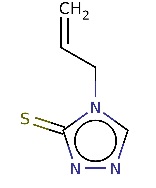 | 65 | 7 | 495.016 | 0.00E+00 | 80.301 |
| 8 | 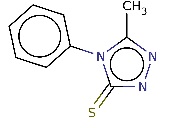 | 143 | 17 | 1075.639 | 0.00E+00 | 72.743 |
| 9 | 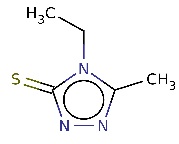 | 60 | 13 | 405.463 | 0.00E+00 | 39.913 |
| 10 | 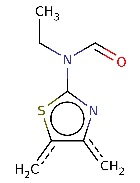 | 188 | 91 | 977.218 | 0.00E+00 | 17.866 |
| 11 | 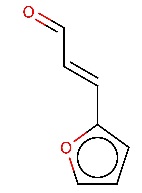 | 420 | 274 | 1883.334 | 0.00E+00 | 13.256 |
| 12 | 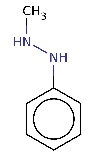 | 394 | 328 | 1522.280 | 0.00E+00 | 10.388 |
| 13 | 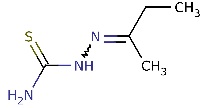 | 60 | 52 | 225.126 | 6.89E-51 | 9.978 |
| 14 | 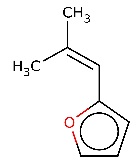 | 159 | 167 | 518.185 | 0.00E+00 | 8.234 |
| 15 | 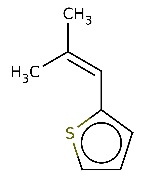 | 50 | 56 | 154.603 | 1.71E-35 | 7.721 |
| 16 | 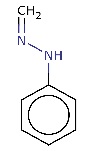 | 614 | 723 | 1827.784 | 0.00E+00 | 7.344 |
| 17 | 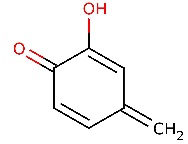 | 197 | 242 | 563.554 | 0.00E+00 | 7.04 |
| 18 | 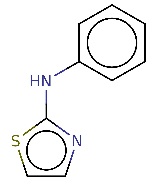 | 162 | 212 | 437.612 | 0.00E+00 | 6.608 |
| 19 | 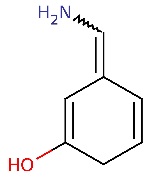 | 147 | 202 | 379.241 | 1.82E-84 | 6.293 |
