# Supplementary material for: Computational Analysis and Predictive Cheminformatics Modeling of Small Molecule Inhibitors of Epigenetic Modifiers
Source: PLoS One. 2016 Sep 13;11(9):e0083032. doi: 10.1371/journal.pone.0083032 (PMC5021286; doi:10.1371/journal.pone.0083032)
Supplement: S3 Table — (DOCX) [file pone.0083032.s003.docx]

**S3 Table:** Shows the significant substructures found in AID 504339 along with their p-value and chi-square statistics.

| **Scaffold No.** | **Scaffold Structure** | **Matches in**  **Actives** | **Matches in**  **Inactives** | **Chi-**  **square** | **P-value** | **Enrichment factor** |
| --- | --- | --- | --- | --- | --- | --- |
| 1 | 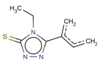 | 110 | 25 | 1755.742 | 0.00E+00 | 88.147 |
| 2 | 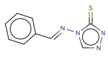 | 118 | 27 | 1880.831 | 0.00E+00 | 87.553 |
| 3 | 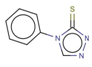 | 277 | 87 | 4095.734 | 0.00E+00 | 63.784 |
| 4 | 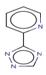 | 96 | 90 | 902.365 | 0.00E+00 | 21.369 |
| 5 | 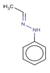 | 228 | 629 | 905.729 | 0.00E+00 | 7.262 |
| 6 | 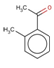 | 248 | 762 | 876.910 | 0.00E+00 | 6.520 |
| 7 | 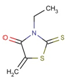 | 119 | 396 | 383.609 | 0.00E+00 | 6.020 |
| 8 | 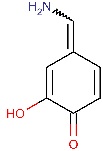 | 96 | 335 | 292.48 | 2.04E-85 | 5.74 |
| 9 | 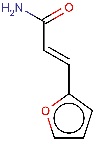 | 135 | 476 | 406.42 | 0.00E+00 | 5.68 |
