# Supplementary material for: Computational Analysis and Predictive Cheminformatics Modeling of Small Molecule Inhibitors of Epigenetic Modifiers
Source: PLoS One. 2016 Sep 13;11(9):e0083032. doi: 10.1371/journal.pone.0083032 (PMC5021286; doi:10.1371/journal.pone.0083032)
Supplement: S4 Table — (DOCX) [file pone.0083032.s004.docx]

**S4 Table:** Shows the enriched substructures in AID 2147 with a threshold of 5 for enrichment factor and p-value less than 0.01.

| **Scaffold No.** | **Scaffold Structure** | **Matches in Actives** | **Matches in Inactives** | **Chi-**  **square** | **P-value** | **Enrichment Factor** |
| --- | --- | --- | --- | --- | --- | --- |
| 1 | 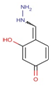 | 53 | 52 | 1383.557 | 0.00E+00 | 54.665 |
| 2 | 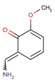 | 56 | 74 | 1231.663 | 0.00E+00 | 40.587 |
| 3 | 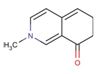 | 65 | 113 | 1192.95 | 0.00E+00 | 30.851 |
| 4 | 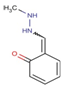 | 168 | 322 | 2879.753 | 0.00E+00 | 27.983 |
| 5 | 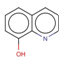 | 85 | 254 | 1021.041 | 0.00E+00 | 17.948 |
| 6 | 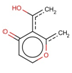 | 38 | 123 | 425.079 | 0.00E+00 | 16.569 |
| 7 | 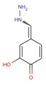 | 44 | 198 | 360.54 | 2.15E-80 | 11.919 |
| 8 | 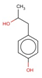 | 35 | 182 | 247.184 | 1.07E-55 | 10.314 |
| 9 | 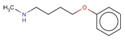 | 33 | 286 | 128.915 | 7.08E-30 | 6.188 |
