# Supplementary material for: Computational Analysis and Predictive Cheminformatics Modeling of Small Molecule Inhibitors of Epigenetic Modifiers
Source: PLoS One. 2016 Sep 13;11(9):e0083032. doi: 10.1371/journal.pone.0083032 (PMC5021286; doi:10.1371/journal.pone.0083032)
Supplement: S5 Table — (DOCX) [file pone.0083032.s005.docx]

**S5 Table:** Shows significantly enriched substructures in AID 540317.

| **Scaffold No.** | **Scaffold Structure** | **Matches in Actives** | **Matches in Inactives** | **Chi-**  **square** | **P-value** | **Enrichment factor** |
| --- | --- | --- | --- | --- | --- | --- |
| 1 | 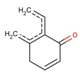 | 81 | 69 | 7442.527 | 0.00E+00 | 201.659 |
| 2 | 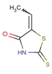 | 28 | 92 | 1080.152 | 0.00E+00 | 52.282 |
| 3 | 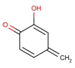 | 43 | 486 | 524.773 | 0.00E+00 | 15.199 |
| 4 | 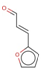 | 66 | 791 | 757.322 | 0.00E+00 | 14.333 |
| 5 | 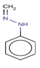 | 116 | 1516 | 1214.422 | 0.00E+00 | 13.144 |
| 6 | 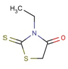 | 42 | 559 | 429.800 | 0.00E+00 | 12.907 |
| 7 | 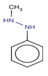 | 40 | 859 | 234.642 | 5.80E-53 | 7.999 |
| 8 | 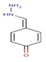 | 31 | 800 | 143.777 | 3.98E-33 | 6.657 |
